# On the Convergence of Ionospheric Constrained Precise Point Positioning (IC-PPP) Based on Undifferential Uncombined Raw GNSS Observations

**DOI:** 10.3390/s131115708

**Published:** 2013-11-18

**Authors:** Hongping Zhang, Zhouzheng Gao, Maorong Ge, Xiaoji Niu, Ling Huang, Rui Tu, Xingxing Li

**Affiliations:** 1 GNSS Research Center, Wuhan University, 129 Luoyu Road, Wuhan 430079, China; E-Mails: hpzhang@whu.edu.cn (H.Z.); zhouzhenggao@126.com (Z.G.); huangling_gnss@whu.edu.cn (L.H.); 2 School of Geodesy and Geomatics, Wuhan University, 129 Luoyu Road, Wuhan 430079, China; E-Mail: lxlq109121@gmail.com; 3 German Research Centre for Geosciences (GFZ), Telegrafenberg, Potsdam 14473, Germany; E-Mails: maorong.ge@googlemail.com (M.G.); turui-2004@126.com (R.T.)

**Keywords:** precise point positioning, convergence time, receiver DCB, global ionosphere delay model (GIM), CMONOC

## Abstract

Precise Point Positioning (PPP) has become a very hot topic in GNSS research and applications. However, it usually takes about several tens of minutes in order to obtain positions with better than 10 cm accuracy. This prevents PPP from being widely used in real-time kinematic positioning services, therefore, a large effort has been made to tackle the convergence problem. One of the recent approaches is the ionospheric delay constrained precise point positioning (IC-PPP) that uses the spatial and temporal characteristics of ionospheric delays and also delays from an *a priori* model. In this paper, the impact of the quality of ionospheric models on the convergence of IC-PPP is evaluated using the IGS global ionospheric map (GIM) updated every two hours and a regional satellite-specific correction model. Furthermore, the effect of the receiver differential code bias (DCB) is investigated by comparing the convergence time for IC-PPP with and without estimation of the DCB parameter. From the result of processing a large amount of data, on the one hand, the quality of the *a priori* ionosphere delays plays a very important role in IC-PPP convergence. Generally, regional dense GNSS networks can provide more precise ionosphere delays than GIM and can consequently reduce the convergence time. On the other hand, ignoring the receiver DCB may considerably extend its convergence, and the larger the DCB, the longer the convergence time. Estimating receiver DCB in IC-PPP is a proper way to overcome this problem. Therefore, current IC-PPP should be enhanced by estimating receiver DCB and employing regional satellite-specific ionospheric correction models in order to speed up its convergence for more practical applications.

## 1. Introduction

Precise Point Positioning (PPP) was firstly proposed by Zumberge *et al.* [1] and a position accuracy of about 2 cm was demonstrated by the Jet Propulsion Laboratory (JPL) with daily dual-frequency data at a single station using precisely estimated satellite orbits and clocks and Earth rotation parameters [1]. Kouba and Heroux verified the PPP technique and confirmed that the positioning accuracy could reach centimeter level using the precise orbits and clocks provided by the International GNSS Service (IGS) [2]. Afterwards, PPP has gained more and more attention due to its cost-efficiency, global coverage and flexibility and became a very useful positioning tool in a number of applications, such as in crustal deformation monitoring (Azua *et al.* [3]), GPS meteorology (Gendt *et al.* [4]), precise orbit determination of low Earth orbit satellites (Bock *et al.* 2003) [5] and high-accuracy kinematic positioning for moving platforms (Gao [6]).

Bar-Sever *et al.* reported the development of the NASA global differential GPS system [7]. Based on the global real-time precise orbit and clock products, PPP was demonstrated to be able to provide real-time kinematic positioning services to meet the requirements of a large number of applications.

Since then, real-time PPP services have been considered a hot topic in GNSS research and development. On the one hand, large efforts have been made to improve the accuracy of the IGS precise orbit and clock products, from 30 cm to 40 cm in the early stages to an optimization of 2.5 cm for orbits and from 1 ns to 2 ns to better than 0.1 ns for clocks (Ye [8]; Geng [9]). On the other hand, PPP itself has been improved steadily. IGS launched its Real-Time Pilot Project (RTPP) aiming at the infrastructure for data collection and communication, the data processing technique and the associated standards for providing such a service. In recent years, IGS has been vigorously promoting real-time GNSS service by operationally providing real-time GNSS orbit and clock products under the frame of RTPP. Nowadays, real-time PPP has become the new focus of future precise positioning services.

One of the major concerns in real-time PPP is that usually it takes about 30 min in order to obtain positions with accuracy better than 10 cm. The PPP positioning accuracy and convergence are mainly influenced by the observing geometry between the station and GPS satellites (Li and Shen [10]), the quality of pseudorange observations and the phase continuity (Teunissen [11]), *etc.* To improve its accuracy and to shorten the convergence time, approaches for PPP ambiguity resolution were developed by estimating the Un-calibrated Phase Delay (UPD) (Ge *et al.* [12]) or mitigating the UPD into satellite clocks (Laurichesse and Mercier [13]; Colinns *et al.* [14]). Although, these approaches were demonstrated to be efficient in accuracy improvement and convergence, it still takes about 10 to 25 min for a reliable fix depending on the quality of the pseudoranges which are needed for resolving wide-lane ambiguities (Geng [9]; Li *et al.* [15]).

As is well known, the first-order ionosphere delay can be eliminated by forming an ionosphere-free observation. Although ionospheric delays in phase and range are expressed by the same ionospheric delay parameter, it is eliminated as different ones for phase and range. Furthermore, the spatial and temporal characteristics of the ionospheric delays and an available a priori correction model, which are implemented as constraints to enhance PPP using single-frequency observations (Beran *et al.* [16]; Shi *et al.* [17]), could not be considered for possible improvement. Juan *et al.* [18,19] developed an enhanced PPP approach where ionospheric model corrections are applied as constraints on the combined ionospheric observations although ionosphere-free observations are used. Alternatively, Li *et al.* proposed PPP using raw GNSS observations with ionospheric parameters with aforementioned constrained and confirmed its improvement on PPP performance in terms of both accuracy and convergence [15]. In this contribution, we investigate the impact of the accuracy of ionospheric delay correction models on PPP performance. The effect of receiver Differential Code Bias (DCB) and its handling are also studied with a large data set.

After a brief introduction of the observation equations, the mathematical model of the ionosphere delay constrained PPP (IC-PPP) is presented, with details on the ionospheric constraints and DCB parameterization. Then, the data processing scenarios are illustrated for assessing the impact of the quality of ionospheric corrections and the effect of receiver DCB and its estimation. Results from a large GPS data set will be presented and discussed.

## 2. Ionospheric Delay Constrained PPP Algorithm

In order to discuss the details of the IC-PPP model, we first introduce the basic GNSS observation equations. Then, an approach to generate satellite-specified ionospheric corrections based on dense regional reference networks is discussed. Of course the temporal and spatial constraints imposed on ionospehric parameters and the DCB parameterization are also presented to complete the IC-PPP algorithm.

### 2.1. Basic Observation Equations

The observation equations of the pseudorange and carrier-phase at frequency band *f_i_* can be expressed as:

(1) $$P_{i}=\rho +c(\delta t_{r}-\delta t^{s})-\rho_{\text{trop}}-{\left(\frac{\lambda_{i}}{\lambda_{1}}\right)}^{2}\rho_{\text{ion},1}-\gamma_{i}(\rho_{\text{dcb}}^{s}+\rho_{r,\text{dcb}})+\Delta +\varepsilon_{P,i}$$

(2) $$L_{i}=\rho +c(\delta t_{r}-\delta t^{s})+\lambda_{i}N_{i}-\rho_{\text{trop}}+{\left(\frac{\lambda_{i}}{\lambda_{1}}\right)}^{2}\rho_{\text{ion},1}+\Delta +\varepsilon_{L,i}$$

where, the units in the equations above are SI units; *c* is the speed of light in vacuum; *s* and *r* represent satellites and receivers, respectively; *P* and *L* are pseudorange and carrier phase observation in length, respectively; *δt_r_* and *δt_s_* represent receiver and satellite clock offset, respectively; ρ denotes the geometry distance between the receiver and satellite; *ρ_trop_* and *ρ_ion_* represent troposphere and ionosphere delays; $\rho_{\text{dcb}}^{s}$ and *ρ_r_*,*_dcb_* are satellite and receiver DCB between pseudoranges at different frequencies, respectively; *γ_i_* is coefficient transforming DCB's effect on pseudorange at frequency *i* based on the satellite clock datum, *i.e.*, $\gamma_{1}=f_{2}^{2}/(f_{1}^{2}-f_{2}^{2})$ for L1 frequency, for L2 frequency $\gamma_{2}=f_{1}^{2}/(f_{1}^{2}-f_{2}^{2})$ if GPS satellite's clock is based on ionosphere-free combination. λ and N represent carrier wavelength and ambiguities, respectively; Δ represents other corrections, including relativity effects, antenna phase center offset, *etc.* and wind-up effect in carrier phase is corrected in advance; *ε_P_* and *ε_L_* are the observation noise of pseudorange and carrier phase, respectively.

In traditional PPP, ionosphere-free phase (LC) and range (PC) based on dual-frequency pesudoranges and carrier phases are used to eliminate the first order ionospheric delay. The residual high order ionospheric delay is usually less than 1% (Hernandez-Pajares *et al.* [19]), which can be ignored in real-time PPP applications. The observation equations of the ionosphere-free combination are as following:

(3) $$\{\begin{array}{c} PC=\frac{f_{1}^{2}}{f_{1}^{2}-f_{2}^{2}}\cdot P_{1}-\frac{f_{2}^{2}}{f_{1}^{2}-f_{2}^{2}}\cdot P_{2}\\ LC=\frac{f_{1}^{2}}{f_{1}^{2}-f_{2}^{2}}\cdot L_{1}-\frac{f_{2}^{2}}{f_{1}^{2}-f_{2}^{2}}\cdot L_{2} \end{array}$$

From Equations (1) and (3), it is very clear that PC and LC are formed independently. In other words, the ionospheric delay parameter in phases is eliminated without considering the range observations with the same ionospheric delay parameter and the same is true for forming the PC observations. This means that the ionospheric parameters in phase and range are treated as different ones and thus it is not equivalent to the elimination of the parameters in a total least square adjustment. By the way, in this combination, the noises of pseudorange and carrier-phase are magnified by a factor of 3.

In order to avoid aforesaid disadvantages of the LC-PPP using Equation (3), IC-PPP is developed where Equations (1) and (2) with the ionospheric delay along the line of sight (LOS) of satellite as unknown parameter are utilized to consider all associated ionospehric constraints in the estimation. The details on the ionospheric constraints and the models will be discussed in the next subsections.

### 2.2. Ionosphere Delay Correction Models

Dual-frequency GNSS observations at ground networks are the basic information for reconstructing ionospheric delay models for both ionosphere study and precise positioning. The ionosphere delay models could be generated at global or regional scales, corresponding to the coverage of the reference networks.

The global model is usually expressed in the form of spherical harmonic functions or grids, for example, the global ionospheric map (GIM) by CODE (Schaer [20]) or by JPL (Mannucci *et al.* [21]). In the global model recovery, it is assumed that the electronic density of the atmosphere is concentrated on a layer at a fixed height, e.g., 350 km. Under this assumption, the slant delays from GNSS observations are expressed by the vertical total electronic content (VTEC) and a mapping function. Then, the coefficients of the spherical harmonic function are estimated to represent the VTEC [20].

For a LOS path of an observed satellite, the position of the ionosphere pierce point (IPP), *i.e.*, the intersection point of the path and the single layer, is computed and then the VTEC at the IPP is calculated using the GIM harmonic spherical coefficients. Then, the VTEC is mapped to slant through a mapping function, for example, the SLMP function (Schaer [22]).

Due to the inaccuracy of the assumption and the mapping function, and the limited station density, the Root Mean Square (RMS) of a global model is usually of about 0.0∼0.9 m (Hernández-Pajares *et al.* [23]) in GPS L1 and varies in different regions. Therefore, ionosphere correction models is also suggested to be constructed based on PPP results of regional reference networks in the form of slant delays of all reference stations to an individual satellite (Tu *et al.* [24]).

**Figure 1.**
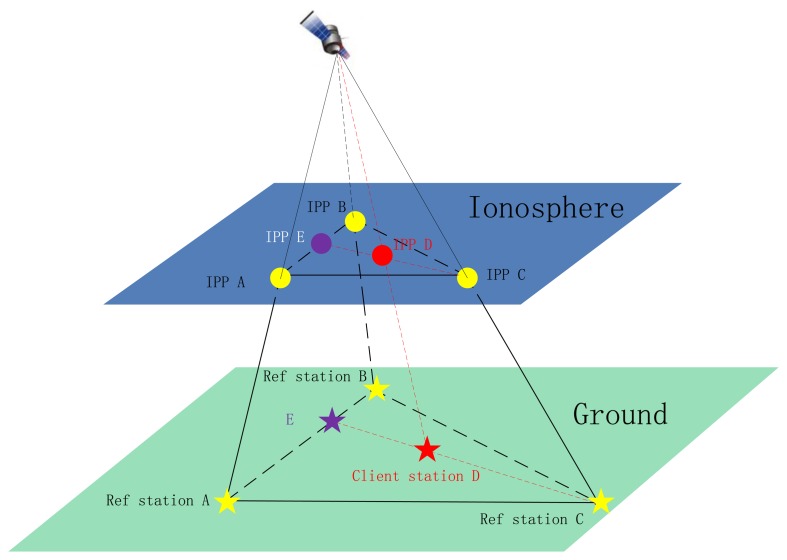
Interpolation of the slant ionospheric delay of a client station using the estimated slant delays of three closest reference stations.

For the regional model, PPP is undertaken for all the reference stations with known coordinates and even receiver DCBs, so that slant ionospheric delays for each LOS can be calculated and serve as ionospheric model. As illustrated in Figure 1, for a LOS of a client receiver, the three closest reference stations are selected and based on their PPP-solved ionospheric delays on the LOS to the same satellite, the ionospheric delays of the client receiver is interpolated according to their geographical locations. It is assessed that there is very slight difference in the interpolated values using coordinates of the ground stations (A, B, C and D in Figure 1) or the IPPs (IPP A, IPP B and IPP C) (Zou *et al.* [25]).

### 2.3. Ionospheric Delay Constraints

First of all, the calculated slant ionospheric delay from an *a priori* model can be imposed as a constraint on the ionospheric parameter of the associated observations. The constraint can be expressed in form of the following pseudo observation equation:

(4) $$v=\rho_{\text{ion},1}-{\tilde{\rho }}_{\text{ion},1},\sigma_{\text{ion}}^{2}$$

where, *ρ̃_ion_*,_1_ and $\sigma_{\text{ion}}^{2}$ are the ionospheric delay calculated from the *a priori* model and its standard deviation (STD), respectively. As is well known, the accuracy of the calculated delays from the global and regional models have quite different quality. Even for the delays from the same model, their accuracy could vary in time and space. Therefore, the STD should be fine-tuned:

(5) $$\sigma_{\text{ion}}^{2}=\frac{1}{\sin^{2}(E)}\cdot \{\begin{array}{cc} \sigma_{\text{ion},0}^{2}+\sigma_{\text{ion},1}^{2}\cdot \cos(B)\cdot & \cos(\frac{t-14}{12}\pi ),8<t<20\text{orB}<60{}^{\circ}\\ \sigma_{\text{ion},0}^{2} & \text{otherwise} \end{array}$$

where E is the satellite elevation; B is the latitude of IPP; *t* is the corresponding local time of the observation epoch at the IPP (0 h∼24 h); $\sigma_{\text{ion},0}^{2}$ is the variance of the zenith delay either given by the model or converted from the VTEC variance which is around 0.4 m for GIM; $\sigma_{\text{ion},1}^{2}$ is also about 0.4 m for tuning the variation of ionospheric delay's variance along latitude and local time.

For the regional model, the STD of the interpolated slant delay can be estimated according to the binterpolation method and the variance of slant ionospheric delay from PPP technique, usually, $\sigma_{\text{ion},0}^{2}$ is about 0.4 m.

In addition to the *a priori* model constraint, the slant ionospheric delays for an individual satellite-receiver pair can be expressed by a stochastic process as follows:

(6) $$\begin{array}{l} \rho_{\text{ion}}(k+1)=\rho_{\text{ion}}(k)+w(k)\\ E(\rho_{\text{ion}}(0))=\rho_{\text{ion}}(0),D(\rho_{\text{ion}}(0))=\sigma_{0}^{2}\\ E(w(k))=0,D(w(k))=q_{\text{ion},t}^{2}(t_{k}-t_{k-1})\\ q_{\text{ion},t}^{2}=\{\begin{array}{ll} \sigma_{\text{ion},0}^{2} & E\geq 30{}^{\circ}\\ \sigma_{\text{ion},0}^{2}/(2\cdot \sin(E)){,}^{2} & E<30{}^{\circ} \end{array} \end{array}$$

where $\sigma_{\text{ion},t}^{2}$ represent the dynamic noise of slant ionospheric delay.

### 2.4. DCB Modeling

Besides the ionospheric delay, the receiver DCB must also be handled differently in LC-PPP and IC-PPP. As usual, satellites DCB must be corrected using the values associated with the clock product. In LC-PPP, the receiver DCB biases all LC ranges by a constant which is absorbed by the receiver clock parameter, therefore, we do not have to consider it. By the way, the DCB of PC measurement is also defined as zero (Dach *et al.* [26]). However, in IC-PPP, DCB has different effect on ranges of different frequency-bands or tracking methods. These effects cannot be compensated by the receiver clock anymore. As is well known, receiver DCB should be estimated as unknown if no precise value is available. Otherwise, both range observations are contaminated and as a consequence convergence will be delayed. In this paper, receiver DCB will be estimated as an unknown parameter in the IC-PPP. As the temporal variations of DCBs are small and in the characteristics of a random process (Wilson *et al.* [27]), receiver DCB can be parameterized with the following equation:

(7) $$x(k)=x(k‐1)+w(k),E(x(0))=x0,D(x(0))=\sigma_{x0,}^{2}E(w(k))=0,D(w(k))=q^{2}(t_{k}‐t_{k-1})$$

where q is the power density of the random process, usually it is about 0.01/ $\sqrt{h}$.

## 3. Experimental Data Processing

In order to evaluate the impact of the quality of ionospheric delay corrections and the receiver DCBs, three PPP modes are employed in the experimental test: PPP using ionosphere-free observations (LC-PPP), PPP using raw observations with ionospheric delay constraints, *i.e.*, ionospheric delays constrained PPP (IC-PPP), and the IC-PPP with receiver DCB parameter (IC-PPP + DCB). The parameters of the three PPP modes are listed in Table 1 and the constraints of the parameters used in the experimental test are also listed in the last column.

With the above-mentioned PPP modes, data from the IGS global network and data from the Crustal Movement Observation Network of China (CMONOC) are processed. For the IGS network, GIM data provided by IGS is used to calculate ionospheric delay correction as constraints in IC-PPP and IC-PPP + DCB, while for the CMONOC network, a reference network is defined for constructing regional ionospheric correction as explained in Section 2.2 and then applied for the client stations as ionospheric constraint for IC-PPP and IC-PPP + DCB. The details of the IGS and CMONOC networks and data sets will be presented Sections 4 and 5, respectively, together with results.

For each of the network, the estimated station positions and convergence time are compared with the known values and against each other, respectively, for assessing their performance. For the IC-PPP and IC-PPP + DCB modes, the estimated ionospheric delays are all interpreted for validating their advantages.

In PPP solutions, the weight of pseudoranges and carrier phases at different elevations are calculated using the following formula (Gendt *et al.* [4]):

(8) $$\text{p}=\left\{ \begin{array}{ll} 1/\sigma_{0}^{2}, & E\geq 30{}^{\circ}\\ 2\cdot \sin(E)/\sigma_{0}^{2}, & E<30{}^{\circ} \end{array}\right.$$

where E is the satellite elevation (the cut off angle is set to 10°), P is the corresponding observation weight; $\sigma_{o}^{2}$ is the observation noise variance. The noises of all the virtual observations are listed in Table 1.

## 4. IGS Data Analysis

**Figure 2.**
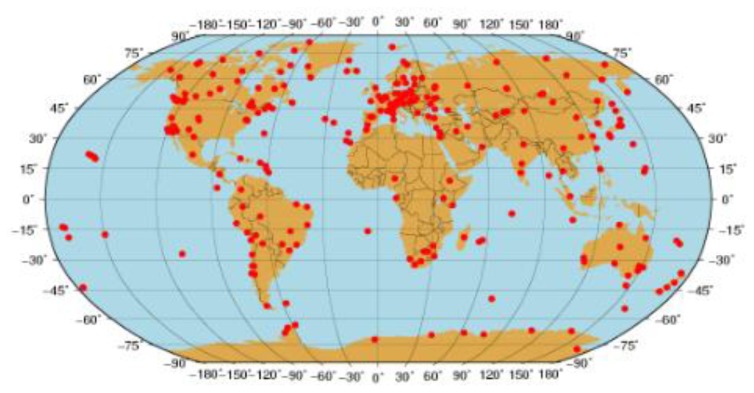
The distribution of the 300 IGS sites used.

For the IGS network, about 300 IGS stations are selected and data from the days 024 to 040, 2012 at the sampling rate of 30 s are processed to evaluate the performance of the three PPP approaches Figure 2 shows the station distribution. In general, there are many more stations in the Northern hemisphere than in the Southern one and quite a few stations in the region close to the equator. Table 2 shows the number of stations in different latitude zones. The GIM is involved in providing *a priori* ionospheric delays for IC-PPP and IC-PPP + DCB.

### 4.1. Static PPP Results

The daily estimated station coordinates of the three processing scenarios are compared with the related IGS weekly solutions. The Root Mean Square (RMS) of the coordinate differences in the NEU is shown in Table 3. From the RMS, the three solutions can achieve very similar position accuracy, about 4 mm, 4 to 9 mm and 14 mm in the north, east and vertical directions, respectively. However, the east component of the IC-PPP without DCB parameter is about 9 mm, significantly larger than that of the other two of about 6 mm. This is most likely due to the neglect of the receiver DCBs.

**Figure 3.**
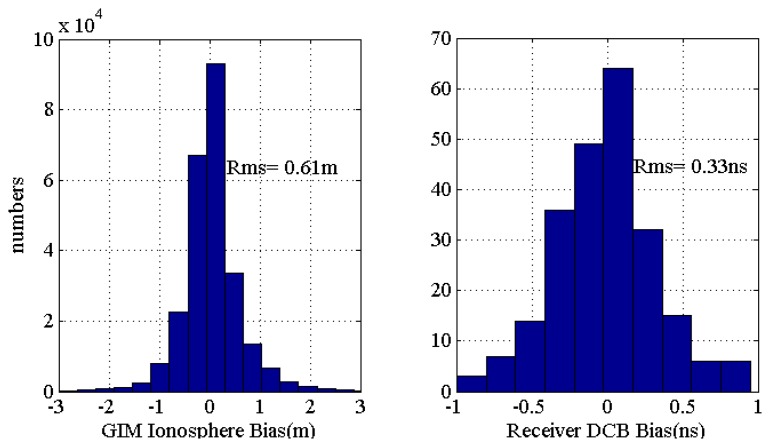
Histogram showing the differences of slant ionospheric delays and receivers' DCB between IGS published results and IC-PPP + DCB derived results at the selected 300 IGS sites.

Figure 3 shows the histogram distribution of the differences between the slant ionospheric delays interpolated from GIM and estimated by the IC-PPP + DCB processing scenario. The RMS of slant ionospheric delays' difference at L1 frequency is 0.61 m, which is about 3.7 TECU. However, GIM, a widely accepted ionospheric delay model, has been fully validated by many technologies and data, and been proved that its RMS is about 2∼8 TECU (Le *et al.* [28]; Hernández-Pajares *et al.* [23]). Therefore the PPP derived ionosphere slant delay is reasonable. As the single layer assumption and the ionosphere mapping function employed in the GIM recovery will certainly limit the resulted model accuracy, the directly estimated slant delays by the IC-PPP + DCB solution should be better than that of GIM.

The differences between IGS and IC-PPP + DCB derived DCBs are shown in Figure 3. The RMS of 0.33 ns confirms an excellent agreement of the estimated DCB with the IGS released ones which fluctuate around 1.0 ns (Hernandez-Pajares *et al.* [29]). In IC-PPP, there is a strong correlation between the ionosphere delay and the receiver DCB, which means that there should be *a priori* information used to constrain receiver DCB or the slant ionospheric delays. However, as Equations (6) and (7) indicate, the mean and dynamic variation characteristic of slant ionospheric delays and receiver's DCB are different from each other, but the derived slant ionospheric delay and DCB results show consistency with IGS. Then, the settings discussed in the section above for IC-PPP + DCB are reasonable, and IC-PPP + DCB is a good solution to invert the slant ionospheric delay.

### 4.2. Kinematic PPP Convergence

**Figure 4.**
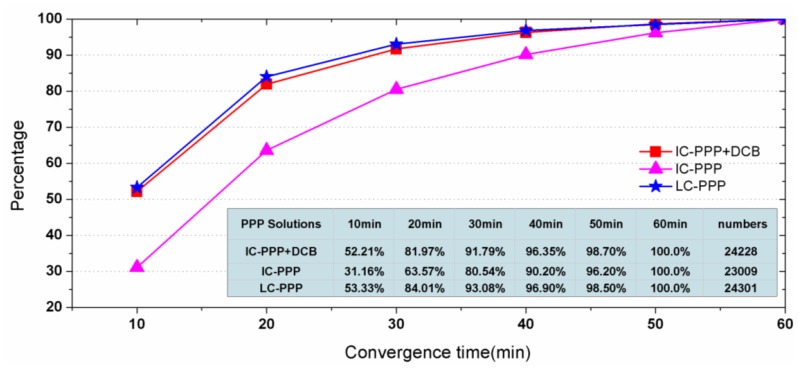
Percentage of PPP results converged to 10cm in horizontal components in different time spans.

To test PPP convergence performance, the daily data are divided into 12 sessions each of two hours. Seven days' data at 300 IGS stations are processed in two-hour sessions, so that a total of 24,521 re-convergence sessions should be involved after removing those sessions missing data. However, there are some sessions that failed in PPP convergence within one hour, and then are removed too. As the last column in Figure 4 shows, in total the valid arcs make up more than 99%.

Figure 4 shows the percentage of the converged sessions along with observing time. Here, a converged session means the accuracy of its horizontal components is better than 10 cm. From Figure 4, within 30 min 91.8%, 80.5% and 93.1% of the sessions converged to 10 cm in horizontal for the processing scenarios IC-PPP + DCB, IC-PPP without DCB parameter, and LC-PPP, respectively.

The larger convergence percentage of IC-PPP + DCB compared with that of the IC-PPP demonstrates that the receiver DCB has a strong impact on IC-PPP convergence. However, it is unexpected that the 93.10% of the LC-PPP solution is slight better than that of IC-PPP + DCB. The possible explanation might be the quality of the GIM is not good enough during this period for mitigating the range noise for better positioning accuracy.

**Figure 5.**
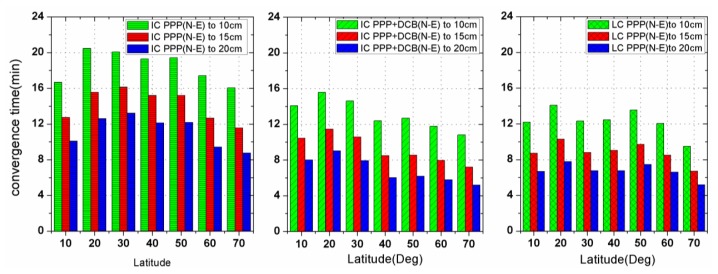
Convergence time for different latitude zones for the three processing scenarios IC-PPP (**Left**), IC-PPP+DCB (**Middle**) and LC-PPP (**Right**). On each sub-plot the convergence green, red and blue bars are for time converged to 10cm, 15cm and 20cm, respectively.

To further study the convergence, the convergence time for stations located in different latitudes are shown in Figure 5 for the three solutions IC-PPP (left), IC-PPP + DCB (middle) and LC-PPP (right). Generally, the left and the middle sub-plots indicate that the convergence time of IC-PPP + DCB accelerates along with the increase of latitude, except for the latitude zone close to the equator where there are rather few stations and the ionosphere equator peaks locate. This trend almost disappears in the right sub-plot where ionospheric delays is eliminated instead of corrected using GIM. Obviously, this trend coincides with the accuracy variations along latitude of GIM too.

**Figure 6.**
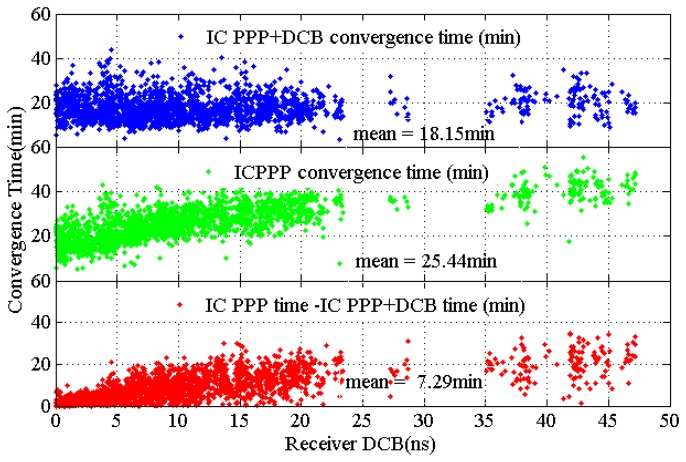
Relationship between receivers DCB magnitude and PPP convergence time in IC-PPP +DCB (**Top**) and IC-PPP (**Middle**) and the differences of their convergence time. The convergence time increases along with the receiver DCB if DCB is not estimated (middle), whereas a unique convergence time is needed for IC-PPP + DCB.

To show the effect of the receiver DCB, the convergence time against the receiver DCB is plotted in Figure 6 for IC-PPP + DCB (top), IC-PPP (middle) and their difference (bottom). The criteria for the time of IC-PPP + DCB and IC-PPP are converging into 10 cm in all three dimensional components.

There is an obvious trend in the IC-PPP where DCB is ignored. It indicates that the convergence time becomes longer as the receiver DCB increases. The trend disappears in the IC-PPP + DCB solution where the receiver DCB is estimated. The mean convergence time improvement from IC-PPP to IC-PPP + DCB is about 7.3 min, nearly 30% as a percentage. As Figure 6 shows, the bigger the receiver DCB, the larger the improvement becomes. The maximum improvement can reach about 50%∼60%. In the case of small receiver DCBs, estimating DCBs will weaken the solution and lead to a longer convergence time which is shown as a negative difference in the bottom panel.

## 5. CMONOC Data Analysis

**Figure 7.**
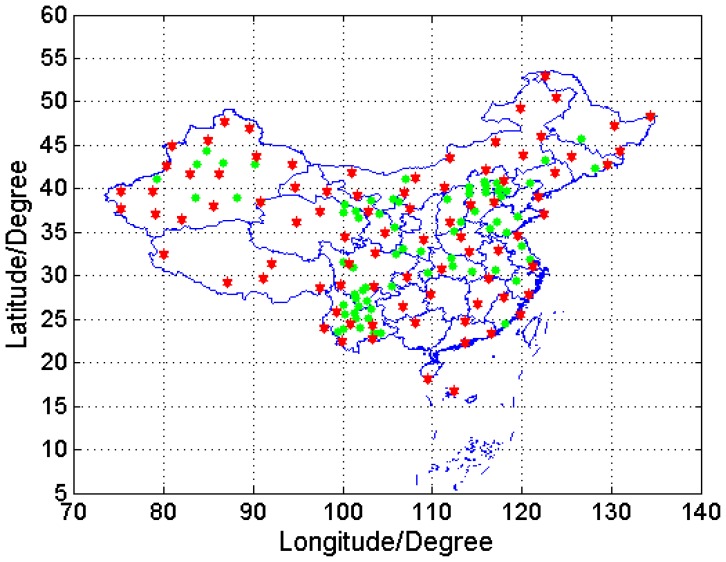
Distribution of the 160 selected CMONOC stations. Red stars represent the reference stations for generating regional ionospheric corrections and green dots indicate PPP test stations.

In order to investigate the impact of the ionospheric correction model on the PPP performance, the Crustal Movement Observation Network of China (CMONOC) is exploited since it can provide more continuous GNSS tracking sites for PPP using regional ionospheric model. There are about 160 stations on DOY 218∼224, 2012 that are selected and divided into two groups: a reference network comprising about 85 stations with a inter-station distance of about 320 km and the others as PPP test stations as shown in Figure 7.

The reference network is used to generate the satellite-specified slant ionosphere delays with the IC-PPP + DCB solution. In the processing, the satellite DCB is calibrated using the IGS products and the station coordinate is fixed to the IGS-like weekly solution. Furthermore, forward and backward filtering are carried out, so that the derived ionospheric delays could achieve an accuracy of better than 2.0 TECU. Then the slant delays at the test stations can be calculated by the linear interpolation of the estimated slant delays of the nearby reference stations. These satellite-specified corrections are referred as to China Regional Model (CRM). Correspondingly, GIM is also used to provide the *a priori* ionosphere delay in the same way as for the aforesaid IC-PPP for IGS stations. To compare their performance, five PPP solutions are carried out, namely, LC-PPP, IC-PPP + DCB using GIM, IC-PPP using GIM, IC-PPP + DCB using CRM and IC-PPP using CRM.

### 5.1. Slant Ionospheric Delays from CMONOC

**Figure 8.**
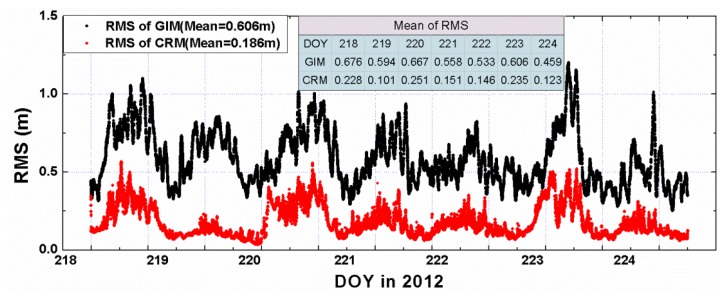
RMS of the residuals of the interpolated slant delays from GIM (black) and CRM (red) with respect to the reference slant delays.

To assess the quality of ionospehric corrections, the data of the 75 test stations is also processed in the same way as for the 85 sites, so that the slant ionospheric delays can be directly estimated from the observations. The estimated delays can be served as reference values to assess the quality of the interpolated corrections from CRM and GIM. As Figure 8 shows, the RMS of the interpolated CRM slant delays is about 0.21 m with respect to the reference values, while the RMS of the GIM derived slant delays is about 0.55 m. This indicates that slant delays from the CRM are generally much more precise than those derived from GIM.

### 5.2. Initial Positioning Results

The initial positioning accuracy of IC-PPP is mainly affected by the level of pseudorange noises, residuals of *a priori* ionosphere delays, and receiver DCB if all other systematic errors are corrected. For LC-PPP, the main factor is the noise of the ionosphere-free pseudorange.

**Figure 9.**
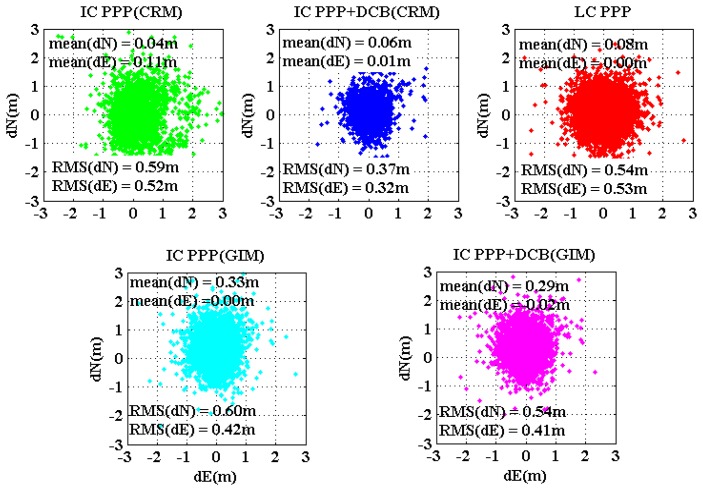
Initial horizontal positioning results for the five PPP schemes. The position of the first three epoches are counted and plotted for all the convergence trials.

**Figure 10.**
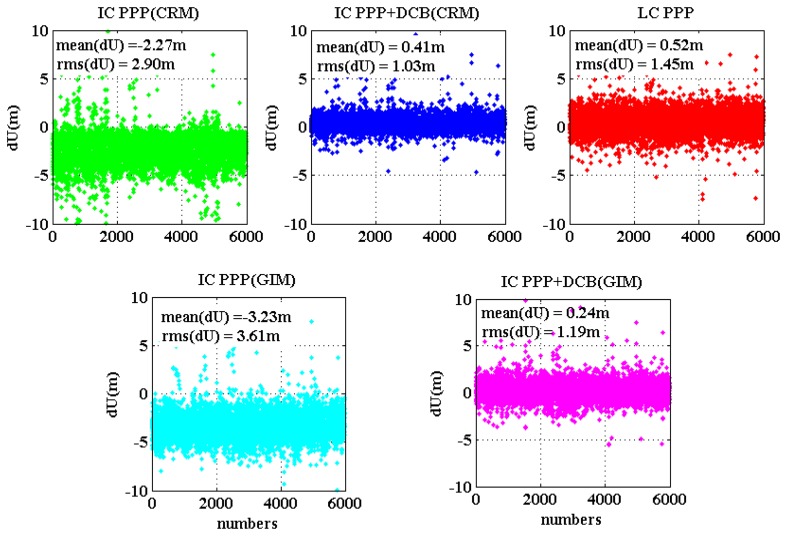
Initial height results for the five PPP schemes. The position of the first three epochs are counted and plotted for all the convergence trials.

The coordinate estimates of the first three epochs of all the test sessions at all sites are compared with the ground true and the residuals are plotted in Figures 9 and 10 for horizontal and vertical components, respectively, and each sub-plot is for one PPP processing mode. Obviously, IC-PPP + DCB using the regional ionospehric model corrections provides the best accuracy both in horizontal and height components, since CRM provides better ionosphere delays than GIM, and receiver DCB is estimated to avoid any possible systematic bias in pseudoranges. As Figure 9 shows, IC-PPP + DCB using CRM and LC-PPP are almost unbiased in the horizontal, but the other three PPP schemes are clearly biased in initial horizontal positioning. In Figure 9, IC-PPP using CRM is biased by around 0.1 m, which is mainly due to the neglect of receiver DCBs, while horizontal components of the IC-PPP using GIM and IC-PPP + DCB using are biased by about 0.3 m. The major reason is most likely due to the poor quality of GIM for which only seven stations over the Chinese territory sites are used.

From Figure 10, the effect of DCB in IC-PPP is mainly on the height accuracy and causes a biased height at the level of about −2 m∼−3 m. If receiver DCB is estimated, as shown in Figure 10, the height bias is decreased within 0.20 m∼0.40 m and the RMS is about 1.0 m, which is at the same level of that of the LC-PPP. This is reasonable since the synthesized noise in IC-PPP is in the level of several decimeters and the VDOP is usually more than 1.0.

### 5.3. Kinematic PPP Convergence

The analysis above shows that the convergence of the IC-PPP + DCB should be faster than that of LC-PPP if the quality of the *a priori* ionosphere corrections is good enough. From the initial positioning analysis, the CMONOC regional dense GNSS network could provide better priori-ionosphere delays for a better convergence for the IC-PPP. The 75 test stations are processed using the above-mentioned five PPP solutions for evaluating their convergence. The data are divided into the two-hour sessions and processed in the same way as for the aforesaid test with IGS stations. Sessions with convergence time longer than 60 min are excluded in the statistics.

**Figure 11.**
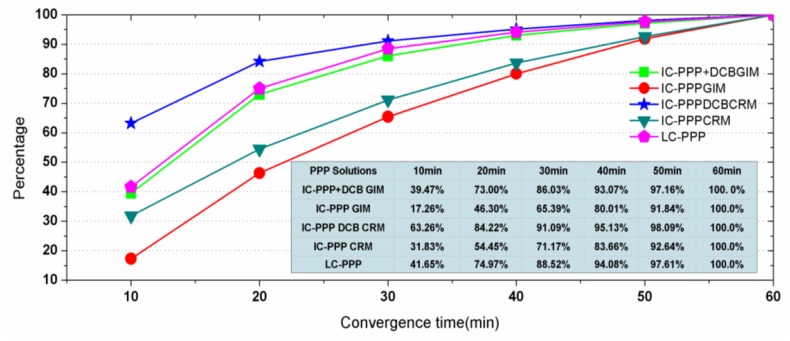
Success rates of convergence into 10 cm in horizontal components of the five PPP schemes.

Figure 11 shows the statistics of the convergence time of the five processing schemes. From the result, the convergence of IC-PPP + DCB using CRM is apparently faster than that of IC-PPP + DCB using GIM and IC-PPP using CRM. Taking the PPP convergence time for position accuracy of 10 cm as an example, as shown in Figure 11, 91% of the sessions can converge within 30 min, 84% within 20 min and 63% within 10 min. The corresponding percentages of the converged sessions for IC-PPP + DCB using GIM are 86%,72%, 39%, and 71%,54% and 32% for IC-PPP using CRM.

**Figure 12.**
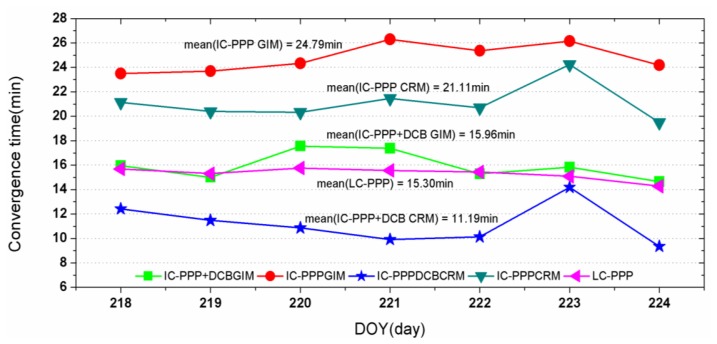
Mean convergence time into 10 cm in both North and East components of five PPP solutions using CMONOC data (minutes).

Figure 12 provides the mean convergence time of the 75 CMONOC stations on the days 218 to 224, 2012. The daily mean convergence time is the average of that of the 900 two-hour sessions of the 75 sites. For IC-PPP + DCB solutions, using CRM could shorten the convergence time, as shown in Figure 12 from 16 min to 11 min compared to that using GIM. For IC-PPP using CRM with DCB parameter, the mean convergence time is greatly reduced from 21 min without the DCB parameter to 11 min. This improvement is under the condition that the receiver DCBs for those 75 sites are almost all around 10 to 12 ns, since all CMONOC receivers are of the same type, Trimble Net R8, while in the IGS network, various types of receivers are deployed and the DCBs are quite different and vary from 0 ns to more than 50 ns, as shown in Figure 6. Comparing IC-PPP + DCB using CRM with LC-PPP, the convergence time could still be shortened from 15 min of the latter one to 11 min. This improvement was not recognized for PPP with IGS stations using GIM presented before because of the limited accuracy of the GIM. As discussed above, the precision of slant ionospheric delays of the regional model is about 0.2 m. Thus, the synthesized noise of IC-PPP + DCB is about $\sqrt{\frac{0.3^{2}}{2}+0.2^{2}}=0.3\text{m}$, which is much smaller than that of LC combination of about 0.8 m. This confirms that the improvement is reasonable.

## 6. Conclusions

The impact of the quality of ionospheric model corrections and receiver DCBs on the convergence of the IC-PPP is investigated through the analysis of a large amount of data. In IC-PPP solution, receiver DCB has significant influence on its convergence. The bigger the DCB, the slower the PPP converges. Estimating receiver DCB in IC-PPP solution is a proper way to overcome the problem. The results, which are derived from 300 IGS sites using GIM as *a priori* ionospheric delays, indicate that the convergence time can be reduced from 254 min to 18 min which is an average improvement of about 28%.

The accuracy of the *a priori* ionosphere delays is also very critical for IC-PPP and IC-PPP+DCB convergence. Regional dense GNSS networks can provide more accurate ionosphere delays than IGS GIM, thus shortening the convergence time. With the CMONOC regional network, the convergence time is reduced from 16 min using IGS GIM to 11 min, which is about a reduction of about 30%.

Therefore, we strongly suggest that receiver DCB should be estimated in current IC-PPP and regional satellite-specific ionospheric correction models should be utilized in order to speed up its convergence for wider applications.

## Figures and Tables

**Table. 1.** Parameter schemes for IC-PPP estimation no italic units.

**Parameters**	**LC-PPP**	**IC-PPP**	**IC-PPP** + **DCB**	**Constraints**	
Position	Static/Kin	Static/Kin	Static/Kin	10 m each component	
Receiver clock	White noise	White noise	White noise	300 m	
Troposphere delay	ZTD	ZTD	ZTD	20 cm + 1 cm/ $\sqrt{h}$	
Receiver DCB	Absorbed	Ignored	Random walk	15 cm + 1 cm/ $\sqrt{h}$	
Ionosphere delay	Eliminated	Slant Delay + Constraint	Slant Delay + Constraint	30 cm + 1 cm/ $\sqrt{h}$
Ambiguities	LC	L1, L2	L1, L2		
Number of observation parameter	N × 2/N + 5	N × 4/3 × N + 5	N × 4/3 × N + 6		

**Table 2.** 300 IGS Sites' distribution at different latitudes.

**Groups**	1	2	3	4	5	6	7
**Latitude (**°**)**	|B| < 10	|B| > 10 |B| < 20	|B| > 20 |B| < 30	|B| > 30 |B| < 40	|B| > 40 |B| < 50	|B| > 50 |B| < 60	|B| > 60
**Numbers**	13	31	32	71	64	45	44

**Table 3.** The overall RMS (in meters) of the coordinate differences of 300 IGS sites in NEU directions of the three processing scenarios.

**PPP Solutions**	**North**	**East**	**Height**
IC-PPP + DCB	0.0038	0.0062	0.0139
IC-PPP	0.0039	0.0091	0.0135
*LC-PPP*	*0.0035*	*0.0053*	*0.0136*

## References

[1] Zumberge J.F., Heflin M.B., Jefferson D.C., Watkins M.M., Webb F.H. (1997). Precise point positioning for the efficient and robust analysis of GPS data from large networks. J. Geophys. Res..

[2] Kouba J., Héroux P. (2001). Precise point positioning using IGS orbit and clock products. GPS Solut..

[3] Azúa B.M., DeMets C., Masterlark T. (2002). Strong interseismic coupling, fault afterslip, and viscoelastic flow before and after the Oct. 9, 1995 Colima-Jalisco earthquake: Continuous GPS measurements from Colima, Mexico. Geophys. Res. Lett..

[4] Gendt G., Dick G., Reigber C.H., Tomassini M., Liu Y., Ramatschi M. (2003). Demonstration of NRT GPS water vapor monitoring for numerical weather prediction in Germany. J. Meteo Soc. Jap..

[5] Bock H., Hugentobler U., Beutler G. (2003). Kinematic and Dynamic Determination of Trajectories for Low Earth Satellites Using GPS. First CHAMP Mission Results for Gravity, Magnetic and Atmospheric Studies.

[6] Gao Y., Shen X. (2002). A new method for carrier-phase-based precise point positioning. Navigation.

[7] Bar-Sever Y., Bell B., Dorsey A., Srinivasan J. (2003). Space Applications of the NASA Global Differential GPS System.

[8] Ye S.R. (2002). Theory and its Realization of GPS Precise Point Positioning Using Un-Differenced Phase Observation.

[9] Geng J., Meng X., Dodson A.H., Ge M., Teferle F.N. (2010). Rapid re-convergences to ambiguity-fixed solutions in precise point positioning. J. Geod..

[10] Li B., Shen Y. (2009). Global navigation satellite system ambiguity resolution with constraints from normal equations. J. Surv. Eng..

[11] Kleusberg A., Teunissen P.J.G. (1996). GPS for Geodesy. Lecture Notes in Earth Science.

[12] Ge M., Gendt G., Rothacher M., Shi C., Liu J. (2008). Resolution of GPS carrier-phase ambiguities in precise point positioning (PPP) with daily observations. J. Geod..

[13] Laurichesse D., Mercier F., Berthias J.P., Bijac J. Real Time Zero-Difference Ambiguities Fixing and Absolute RTK.

[14] Collins P., Lahaye F., Heroux P., Bisnath S. Precise Point Positioning with Ambiguity Resolution Using the Decoupled Clock Model.

[15] Li X., Ge M., Zhang H., Wickert J. (2013). A method for improving uncalibrated phase delay estimation and ambiguity-fixing in real-time precise point positioning. J. Geod..

[16] Beran T., Kim D., Langley R.B. High-Precision Single-Frequency GPS Point Positioning.

[17] Shi C., Gu S., Lou Y., Ge M. (2012). An improved approach to model ionospheric delays for single-frequency precise point positioning. Adv. Space Res..

[18] Juan J.M., Sanz J., Hernández-Pajares M., Samson J., Tossaint M., Aragón-Ángel M., Salazar-Hernández D.J. (2012). Wide area RTK: A satellite navigation system based on precise real-time ionospheric modeling. Radio Sci..

[19] Juan J.M., Hernández-Pajares M., Sanz J., Ramos-Bosch P., Aragon-Angel A., Orus R., Ochieng W., Feng S., Jofre M., Coutinho P. (2012). Enhanced precise point positioning for GNSS users. IEEE Trans. Geosci. Remote Sens..

[20] Schaer S., Gurtner W., Feltens J. IONEX: The Ionosphere Map Exchange Format Version 1.

[21] Mannucci A.J., Wilson B.D., Yuan D.N., Ho C.H., Lindqwister U.J., Runge T.F. (1998). A global mapping technique for GPS-derived ionospheric total electron content measurements. Radio Sci..

[22] Schaer S. (1999). Mapping and predicting the earth's ionosphere using the global positioning system. Geod. Geophys. Arb. Schweiz.

[23] Hernández-Pajares M., Juan J.M., Sanz J., Orus R., Garcia-Rigo A., Feltens J., Komjathy A., Schaer S.C., Krankowski A. (2009). The IGS VTEC maps: A reliable source of ionospheric information since 1998. J. Geod..

[24] Tu R., Ge M., Zhang H., Huang G. (2013). The realization and convergence analysis of combined PPP based on raw observation. Adv. Space Res..

[25] Zou X., Tang W., Shi C., Liu J. (2012). A New Ambiguity Resolution Method for PPP Using CORS Network and its Real-Time Realization. China Satellite Navigation Conference (CSNC) 2012 Proceedings.

[26] Dach R., Hungentobler U., Fridez P., Michael M. (2007). Bernese GPS Software Version 5.0.

[27] Wilson B.D., Mannucci A.J. Instrumental Biases in Ionospheric Measurement Derived from GPS Data.

[28] Le A.Q., Tiberius C.C.J.M., van der Marel H., Jakowski N. (1998). Use of Global and Regional Ionosphere Maps for Single-Frequency Precise Point Positioning. Observing our Changing Earth.

[29] Petrie E.J., Hernández-Pajares M., Spalla P., Moore P., King M.A. (2011). A review of higher order ionospheric refraction effects on dual frequency GPS. Surv. Geophys..

